# Hierarchical compression of *Caenorhabditis elegans* locomotion reveals phenotypic differences in the organization of behaviour

**DOI:** 10.1098/rsif.2016.0466

**Published:** 2016-08

**Authors:** Alex Gomez-Marin, Greg J. Stephens, André E. X. Brown

**Affiliations:** 1 Champalimaud Neuroscience Programme, Champalimaud Centre for the Unknown, Lisbon, Portugal; 2 Behavior of Organisms Laboratory, Instituto de Neurociencias CSIC-UMH, Alicante, Spain; 3 Department of Physics and Astronomy, Vrije Universiteit Amsterdam, Amsterdam, The Netherlands; 4 Okinawa Institute of Science and Technology, Okinawa, Japan; 5 MRC Clinical Sciences Centre, London, UK; 6 Institute of Clinical Sciences, Imperial College London, London, UK

**Keywords:** *Caenorhabditis elegans*, behaviour, genetics

## Abstract

Regularities in animal behaviour offer insights into the underlying organizational and functional principles of nervous systems and automated tracking provides the opportunity to extract features of behaviour directly from large-scale video data. Yet how to effectively analyse such behavioural data remains an open question. Here, we explore whether a minimum description length principle can be exploited to identify meaningful behaviours and phenotypes. We apply a dictionary compression algorithm to behavioural sequences from the nematode worm *Caenorhabditis elegans* freely crawling on an agar plate both with and without food and during chemotaxis. We find that the motifs identified by the compression algorithm are rare but relevant for comparisons between worms in different environments, suggesting that hierarchical compression can be a useful step in behaviour analysis. We also use compressibility as a new quantitative phenotype and find that the behaviour of wild-isolated strains of *C. elegans* is more compressible than that of the laboratory strain N2 as well as the majority of mutant strains examined. Importantly, in distinction to more conventional phenotypes such as overall motor activity or aggregation behaviour, the increased compressibility of wild isolates is not explained by the loss of function of the gene *npr-1*, which suggests that erratic locomotion is a laboratory-derived trait with a novel genetic basis. Because hierarchical compression can be applied to any sequence, we anticipate that compressibility can offer insights into the organization of behaviour in other animals including humans.

## 1. Introduction

In introducing his four questions of ethology [1], Tinbergen emphasized that observation shapes how mechanistic and evolutionary questions are answered. That is, what we choose to measure determines the causal units that will form our explanations. By this reasoning, exploring new ways of quantifying behaviour may identify new phenomena that were not apparent in previous representations. These new phenomena can then become the subject of mechanistic studies to dissect their genetic or neural implementation. The importance of understanding how animals structure their behaviour was recognized in part by the example set by genetics [2], in which many of the principles of inheritance were elucidated through careful observation and experimentation long before the physical nature of genes was known. For animal behaviour, the analogous long-term goal is to generate or constrain hypotheses on the genetic and neural control of behaviour from the structure of behaviour itself.

Advances in automated imaging and computer vision make it possible to revisit the question of behavioural representation without relying on expert annotation. These methods have been used directly for quantitative phenotyping to measure behavioural differences in response to genetic and neural perturbation [3–11], as well as to study the dimensionality, dynamics and structure of animal behaviour [12–16]. However, even with the latest technology, automated analysis in complex natural environments remains challenging [17]. Instead, we study the full structure and complexity of a behavioural repertoire in a simpler environment and focus on the spontaneous crawling of the nematode worm *Caenorhabditis elegans* confined to the two-dimensional surface of an agar plate. We have recently introduced a discrete representation of crawling postures and used it to identify short behavioural motifs that worms use to respond to sensory stimulation or that differ between worm strains isolated from different parts of the world [18]. Here, we explore whether data compression algorithms, which have been applied in domains where discrete data are common such as natural language processing and genomics, can reveal structure in worm locomotion.

Our approach is based on the minimum description length principle, which states that the best model is the one that describes the data most concisely [19]. We apply the minimum description length principle to behaviour by first constructing a dictionary of elementary behavioural states and then merging these states into longer sequences using a data compression algorithm. The resulting new dictionary then serves as the ‘model’ of the behavioural data. Repeated steps of compression can find patterns and ‘patterns of patterns’ in behaviour as proposed by Dawkins [2], thus generating a hierarchical representation of behavioural data. In addition, the degree to which these steps reduce the total length of the sequence and dictionary, the compressibility, offers a quantitative, objective measure of the behavioural complexity.

The connection between iterated dictionary compression and hierarchical organization allows us to pursue two goals at once: to achieve maximum compression of the data and to mine its structure for biological meaning. In *C. elegans*, we find that the dictionary sequences resulting from the compression algorithm represent rare but relevant behavioural motifs. We also measure the compressibility of behaviour and find that worm locomotion has intermediate compressibility poised between random and repetitive and that wild isolates of *C. elegans* have locomotion that is more ordered than the laboratory reference strain N2 and mutants in an N2 background.

## 2. Experiments

The data analysed in this paper come from two previous studies [4,18]. All N2, wild-isolate and mutant tracking on food was done using single worms that were picked to the centre of a spot of *Escherichia coli* OP50 on a 25 mm agar plate. Worms were allowed to habituate for 30 min before being tracked for 15 min. The worm side (whether it was on its left or right side) was manually annotated, using a stereomicroscope, before transferring plates to the tracking microscope. For off-food and chemotaxis experiments, worms were picked to the centre of 55 mm agar plates and recorded immediately. The attractant for chemotaxis experiments was 1 µl of benzaldehyde (diluted 1 : 100 in EtOH).

## 3. Behavioural analysis

### 3.1. Posture discretization and time warping

**Figure 1.**
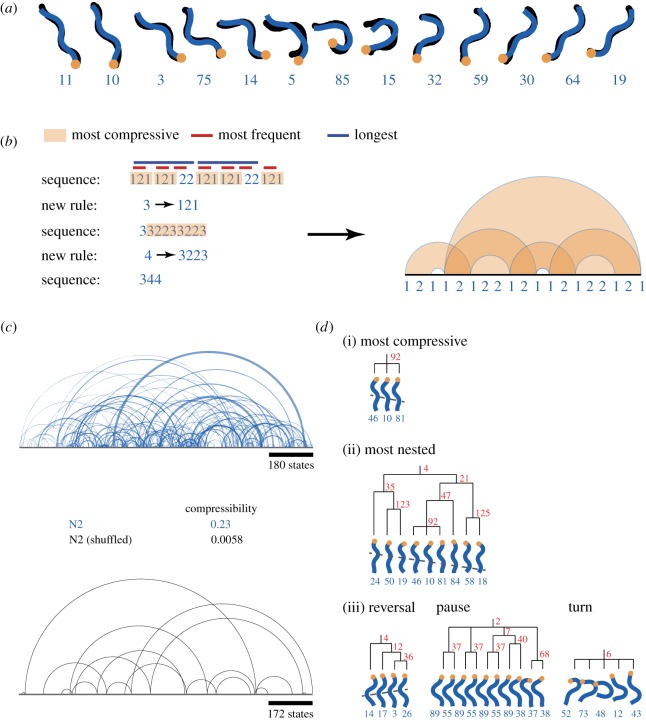
Dictionary-based compression extracts hierarchical structure in posture sequences. (*a*) Locomotion is represented as a sequence of discrete postural states. At each point in time, the original skeleton (black) is matched by its nearest-neighbour posture in a set of 90 template postures. The orange dot indicates the head. The numbers beneath each shape are the labels of the template postures in each case. (*b*) Simple sequence to illustrate the compressive algorithm. For the indicated sequence, the subsequence that results in the greatest compression when it is replaced by a new state label is {1, 2, 1}. In the second iteration {3, 2, 2, 3} and {3, 3, 2, 2} are equally compressive. We simply take the sequence that occurs first in the sorted list of unique sequences. The arc diagram on the right connects adjacent repeats of dictionary sequences. (*c*) An arc diagram for a sequence of worm locomotion (blue) and the corresponding arc diagram for the same sequence following random shuffling (black). (*d*) Selected c-grams discovered from 150 min (approx. 10^4^ postures) of worm behaviour. The most compressive sequence (i), the most nested c-gram (ii) and three other behaviours (iii) are plotted underneath dendrograms that show the hierarchical structure represented in the dictionary. The numbers in red indicate the number of times that the sequence under each branch occurred in the 150 min. (Online version in colour.)

The angles of the worm midlines were determined at 49 equally spaced points [16]. The continuously varying skeleton angles were then discretized by matching the posture in each frame to its closest match in a set of 90 postural templates that were derived from wild-type N2 worms using *k*-means clustering (figure 1*a*). For details on the clustering and discretization, see [18]. Because the motion of the worm between frames is often smaller than the difference between the 90 template postures, this procedure leads to the same template being fitted in several consecutive frames. In order to recognize repeated behaviours performed at different speeds, we use a simple non-uniform time warping: repeats are removed from the posture sequences (for example, the sequence {1, 2, 3, 1, 1, 1, 4, 1} would be reduced to {1, 2, 3, 1, 4, 1}). Nevertheless, temporal information is not lost, because we record the duration of each sequence template for subsequent analysis. In order to compare results across mutant strains and conditions, we used the same wild-type posture templates in all cases.

### 3.2. Compression algorithm

Many popular dictionary compression algorithms are designed to work ‘online’ with little memory and to scan the data from left to right in a single-pass looking for repeated patterns. We are more interested in finding repeating patterns than in single-pass memory-efficient compression, and so we use an ‘offline’ algorithm that considers the entire sequence at each iteration. We follow Nevill–Manning and Witten's offline ‘compressive’ heuristic for inferring hierarchies of repetitions in sequences [20]. This is a dictionary compression method in which repeated subsequences are added to a dictionary and replaced by a new symbol that indicates where the subsequence is contained in the dictionary. At each iteration, the subsequence that is replaced is the one that gives the maximal compression taking into account its length and frequency as well as the size of the dictionary. The savings, *S*, owing to replacing a subsequence are given by *WN* − (*W* + 1 + *N*), where *W* is the length of the subsequence and *N* is the number of times it occurs in the sequence that is being compressed. The first term is the reduction in the length of the sequence while the second term includes the increase in the size of the dictionary (*W* + 1) and the number of new symbols introduced in the compressed sequence (*N*). In the case of ties, where two subsequences are equally compressive, the subsequence that appears first in the sorted list of unique subsequences is replaced and added to the dictionary. This procedure is applied recursively until no more compressive repeats are found. Note that the compression algorithm is lossless. The original sequence can be exactly recovered using the compressed sequence and corresponding dictionary. The algorithm is also greedy, taking the locally most compressive sequence at each iteration, and thus is not guaranteed to find the globally most compressive dictionary. See the electronic supplementary material, figure S1, for an extended example explaining how the sequence in figure 1 is processed.

For faster computation, we calculate *S* only for subsequences up to length *W*_max_. We used *W*_max_ = 10 for the results presented here. Increasing *W*_max_ to 15 gave identical results in 97% of cases in a test of 200 worms and, where results differed, the difference in compressibility was small (electronic supplementary material, figure S2). The compressibility of a sequence of uncompressed length *l* is given by the sum of the savings *S* at each iteration divided by *l*.

## 4. Results

### 4.1. Hierarchical compression of posture sequences identifies behavioural structure

Dictionary-based compression relies on an ability to identify repeated patterns in a symbolic sequence. Worm locomotion can be converted to such a symbolic sequence by representing the continuously varying worm body shape as a sequence of discrete postures (figure 1*a*). In this representation, the original skeleton (in black) is matched by its nearest-neighbour posture in a set of 90 template postures (in blue) at each point in time. The templates themselves are determined using *k*-means clustering, with *k* = 90 postures chosen to capture most of the variance of worm shapes (approx. 80%) without being overly complex [18]. Approximately repeated behaviours can now be found simply by identifying repeated symbolic sequences or *n*-grams. An *n*-gram is any subsequence of symbols of length *n*.

In a dictionary-based compression algorithm, a sequence is compressed by adding an *n*-gram to a dictionary and replacing each instance of that *n*-gram in the original sequence by a new 1-gram not previously present in the sequence. Maximal compression is achieved for *n*-grams that are both long and frequent. We call these maximally compressive patterns ‘c-grams' to distinguish them from the larger set of *n*-grams they are drawn from. This is illustrated for a simple sequence with two symbols in figure 1*b*. In this example, we save seven symbols in total, because the original sequence was 19 symbols long, the compressed sequence is 3 and the dictionary contains a total of nine symbols. The compressibility per symbol is therefore 7/19, or 37% of the original sequence length (see the electronic supplementary material, figure S1, for a more detailed explanation).

To visualize the replacement rules for a given sequence, we plot an arc diagram that connects each neighbouring c-gram that was used in constructing the dictionary (figure 1*b*, right). Frequently occurring c-grams are thus connected by short arcs, whereas rarely occurring behaviours are connected by longer arcs. The width of the arc corresponds to the length of the c-grams that are connected. When applied to wild-type worm locomotion (figure 1*c*), the arc diagram clearly shows that the majority of c-grams are frequent and short (small, thin arcs) but that there are some that are relatively rare and are separated by a long distance (longer arcs). The longest arcs connect c-grams that were only observed twice in the entire 1700 state sequence. This structure does not merely reflect chance repeats owing to the finite number of symbols (the labels of the 90 template postures). When the sequence is randomly shuffled to maintain the posture frequencies but destroy temporal order, very few repeats are observed (figure 1*c*, bottom).

Notably absent in the arc diagrams of worm behaviour are long and highly nested repeats, which would be seen if worms perfectly repeated long sequences at different times. To provide further intuition for the level of repetition seen in spontaneous locomotion, we use the same algorithm to compress texts with increasing levels of structure and repetition: *Moby Dick* by Herman Melville (as used in a previous study on finding motifs in unannotated strings [21]), *The Raven* by Edgar Allan Poe, and *Shake it Off* by Taylor Swift (electronic supplementary material, figures S3 and S4).

Some illustrative c-grams derived from a 30 min (two 15 min sequences concatenated) sample of wild-type locomotion on food are shown in figure 1*d*. The most compressive sequence overall is shown at the top (i). This is the subsequence selected by the compression algorithm as providing maximum compression of the original sequence in the first iteration. Therefore, by construction, it is always ‘simple’ in the sense that it has no nesting structure. In this case, it is a short bout of forward locomotion, consistent with expectations given that wild-type worms spend a significant portion of the time crawling forwards with a stereotyped gait.

The most nested c-gram found in the 30 min is shown in the middle (ii). Note that it contains the most compressive sequence from (i). Two suffixes are added (posture 84 appeared after the most compressive subsequence 47 times and this 4-gram was found with the 2-gram {58, 18} 21 times in a later iteration). Finally, a prefix completes the larger behavioural unit. This illustrates how units formed by basic templates are re-used within larger units. In addition to various kinds of forward locomotion, we find c-grams corresponding to other behaviours. An example of a reversal, pause and a turn are shown at the bottom of figure 1*d*(iii).

### 4.2. Compressive sequences increase discriminative power across environmental conditions

Previous analysis has shown that the frequencies of 3-grams used by worms during locomotion can be used to characterize behavioural differences in different environments (on a lawn of bacterial food, on an agar plate without food and during chemotaxis towards an attractant) [18]. However, it is possible that sequences of other lengths are more informative for comparisons of behaviour in different conditions. The c-grams in the dictionary produced by hierarchical compression have variable lengths that are chosen adaptively based on the input data, in contrast to the fixed-length approach based on 3-grams. To determine whether they are relevant for behavioural comparisons, we re-analysed the data for worms in the different conditions.

**Figure 2.**
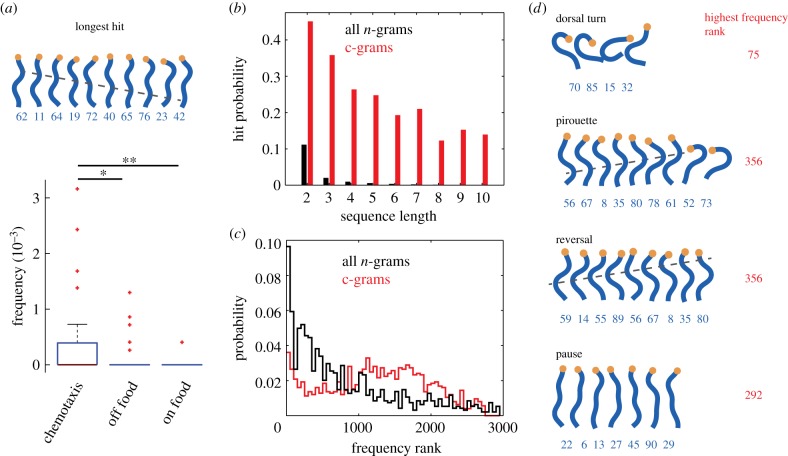
c-grams are rare but relevant subsequences. Hits are any sequences that are found to have a different frequency between N2 animals crawling on food, off food or performing chemotaxis. (*a*) The longest hit is a bout of forward locomotion that is more common during chemotaxis. The box plot shows the frequency of this behaviour in the three conditions (red points are outliers, which are greater than the difference between the 25th and 75th percentiles outside of the box). (*b*) In each condition, the most compressive sequence is a hit in at least one comparison, indicating that compressive sequences are more likely to be modulated across conditions than *n*-grams as a whole. (*c*) The c-gram hits are more evenly spaced across the frequency distribution than those found using all *n*-grams. (*d*) Canonical worm behaviours are identified through compression and these would be missed by focusing only on the most frequently occurring *n*-grams. The behaviours are shown on the left with their highest frequency rank observed across all worms in the comparison group shown in red to the right. (Online version in colour.)

We first compressed the postural sequences of each worm in each condition to produce a dictionary of c-grams for that individual. We then pooled all of the c-grams across all conditions, keeping only the unique c-grams, and compared the distributions of c-gram frequencies between conditions using rank-sum tests, adjusted for multiple comparisons to control the false discovery rate at 5%, using the Benjamini–Yekutieli procedure [22]. Any sequence that was found to have a significantly different frequency between at least two conditions is a ‘hit’. The longest hits we detected were 10 postures long and represented bouts of forward locomotion. This was not because the maximum sequence length considered in a single iteration was 10 (see section Behavioural analysis), because the same six sequences were still the longest hits when the maximum sequence length was increased to 15. One of the six 10-posture hits is shown in figure 2*a* and represents a persistent bout of forward locomotion which is most common during chemotaxis towards an attractant.

The finding that c-grams with lengths up to 10 can be used to show behavioural modifications between conditions motivated us to revisit the previous analysis, using *n*-grams with lengths of up to 10. For this relatively small dataset consisting of 115 worms recorded for 15 min, this was tractable, but still required the consideration of 1.02 million unique *n*-grams. Of these, only 0.2% are used with a significantly different frequency in at least one of the conditions and this percentage is lowest for the longest sequences (figure 2*b*). In contrast, there were only 3014 unique c-grams in the entire pool, with 30% being significantly modulated in the environmental conditions. Furthermore, the fraction of significantly modulated behaviours remains high up to the maximum hit length. The *n*-gram hits are more likely to come from relatively frequent *n*-grams, whereas the c-gram hits are spread more evenly across the frequency spectrum (figure 2*c*). The precise frequencies and compressibilities change with the number of postures used in the representation, but the overall conclusions do not depend sensitively on the number of postures (electronic supplementary material, figure S5).

It could be that the hierarchical compression algorithm is simply selecting frequent behaviours and that these are more likely to be informative for comparing worms in different conditions. To check this, we repeated the *n*-gram analysis, but took only the five most frequent *n*-grams of each length up to 10 from each worm and added it to the pool. This resulted in 3905 unique *n*-grams to use for comparing worms in the different conditions. This improved the efficiency of hit detection, in fact increasing it above that of the c-grams, especially for short *n*-grams (electronic supplementary material, figure S6*a*). However, this improvement in efficiency comes at a cost: rare behaviours are no longer included in the analysis. This can be seen directly in the frequency rank distribution of the hits (electronic supplementary material, figure S6*b*). Because this distribution includes the rank of each hit across all individuals, it is possible, in principle, that some of the *n*-grams that are among the five most frequent in one individual would be extremely rare in another individual, especially in a different condition. For the data considered here, that is not the case. The frequent *n*-gram distribution shows a much steeper drop-off than the c-gram distribution.

Examples of rare hits that would have been missed by focusing only on the most frequent *n*-grams are shown in figure 2*d*. These include potentially interesting behaviours such as a dorsal turn, a pirouette (reversal followed by turn) and a long reversal. Their highest rank across all individuals is shown in red for each behaviour.

### 4.3. Worm behavioural sequences have intermediate compressibility

**Figure 3.**
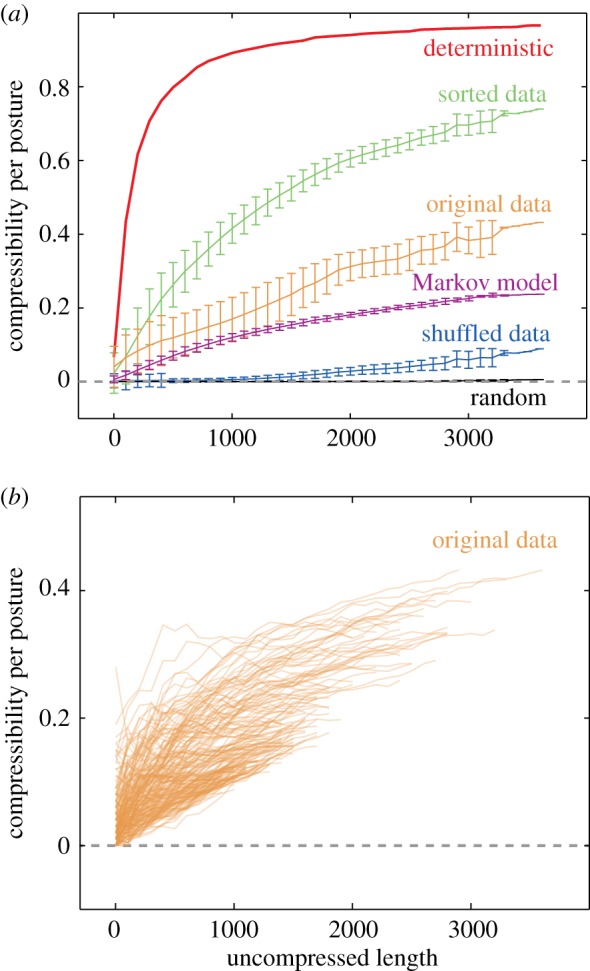
Worm locomotion sequences are poised between random and deterministic, which leads to intermediate compressibility. (*a*) The compressibility per posture increases as a function of length for N2 locomotion sequences (orange). Uniform random sequences with 90 states (black) and a deterministic sequence consisting of 1–90 repeated (red) provide lower and upper bounds on compression. Shuffled (blue) and sorted (green) sequences provide related bounds constrained by having the same posture probability distributions as the observed locomotion sequences. A Markov chain simulated using the observed posture transition probabilities provides a more realistic model of locomotion sequences. (*b*) Compressibility as a function of length for individual worms shows the variability in compressibility. Many of the least compressible individuals have shorter uncompressed lengths, indicating that these worms moved less (had fewer posture transitions) during the 15 min they were recorded. (Online version in colour.)

Hierarchical compression provides a new global feature for characterizing worm behaviour: the compressibility of the sequences. It is clear that highly repetitive sequences will be more compressible than random sequences and we know from the plot in figure 1*c* that worm behavioural sequences are not random. We also know that compressibility must be greater than 0 and less than 1 by definition. To provide further intuition, we compared the compressibility of several ‘toy’ sequences (simulated controls) with real worm behavioural sequences as a function of sequence length (figure 3*a*).

The first toy sequence we considered was a deterministic sequence that is simply the symbols 1–90 repeated in turn up to the desired length. This sequence is highly compressible, surpassing 0.8 compressibility for sequence lengths below 1000. Compressibility increases with length as more nearly optimal sequences are found, but can only reach 1 in the limit of infinite sequences. At the other extreme, we considered random sequences generated by sampling values from 1 to 90 from a uniform distribution. Uniform random sequences with 90 possible symbols are essentially incompressible for all observed lengths. At a length of 1000, the compressibility is 1 × 10^−4^ ± 3 × 10^−4^ (mean ± s.d.). In contrast, behavioural sequences from wild-type N2 worms crawling on food show intermediate compressibility, reaching 0.4 for the longest sequences considered. Control sequences that are more similar to real behaviour sequences were also generated by sorting and randomly shuffling behaviour sequences, yielding sequences that are more and less ordered than the original sequences but that have the same posture frequencies. Again, the natural sequences are poised between random and ordered. Finally, we also compared the natural sequences with sequences generated from a first-order Markov model with transition probabilities determined from worm behaviour sequences. Although more similar than shuffled sequences, the Markov model sequences are still less compressible (i.e. less stereotyped) than the original worm sequences.

Plots of compressibility as a function of length for individual worms reveal interworm variability in compressibility (figure 3*b*). The least compressible worm sequences are also among the shortest, which result from worms that move less and therefore have fewer transitions. The fact that shorter sequences are more random suggests that the shape transitions that drive locomotion are more stereotyped than those that occur during dwelling. As expected, decreasing the number of postures in the representation increases compressibility (more repetition) and increasing the number of postures decreases compressibility (less repetition; electronic supplementary material, figure S7).

### 4.4. Stereotypy varies across strains and does not simply reflect the degree of locomotion

Compressibility is a distinct feature for comparing the stereotypy of worm behaviour and so we analysed data from previously published mutant strains [4] and wild isolates [18]. Compressibility per symbol increases with length (figure 3), because there are more opportunities for compressive subsequences to be found in longer sequences. We therefore chose to compare worms using a fixed sequence length of 500. Sequences from worms that went through more than 500 distinct postures were divided into 500-posture chunks for analysis. Because dwelling worms seem to be less stereotyped than roaming worms (figure 3*b*), we also kept a track of the time worms spent in each posture.

**Figure 4.**
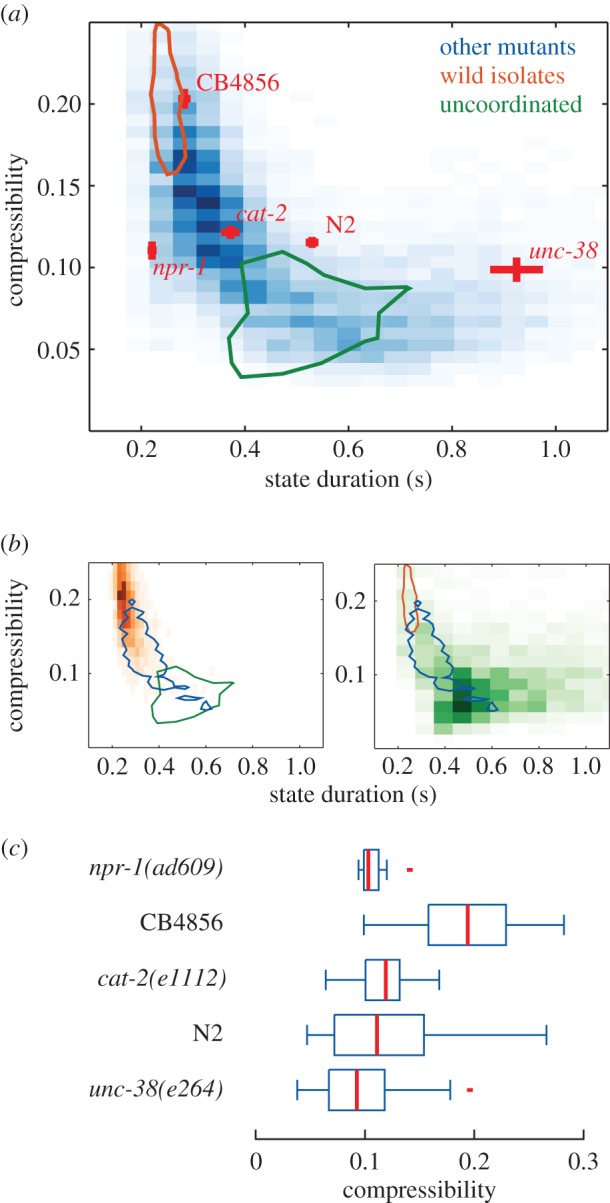
Wild-isolate locomotion is more stereotyped than that of most mutant strains. (*a*) Two-dimensional histogram of the distribution of compressibility against postural state duration for a set of 239 mutant strains that are not uncoordinated (‘other mutants’). The red bars show the mean ± s.e., for a selection of strains. The contours show the extent at half-maximum of the distributions for 18 wild isolates (orange) and 63 uncoordinated mutants (green). The wild-isolate and uncoordinated distributions are plotted separately in (*b*). (*c*) Box plots show the compressibility measured on 500-posture chunks for the strains highlighted in (*a*). CB4856 is more compressible than either N2 (*p* = 4.7 × 10^−8^) or *npr-1(ad609)* (*p* = 3.3 × 10^−5^) using a rank-sum test. (Online version in colour.)

In figure 4*a*, we show a two-dimensional histogram of the distribution of compressibility against state duration for a set of 239 mutant strains that are not uncoordinated (‘other mutants’). Each point in the histogram comes from one 500-posture chunk. The state duration value is simply the average time spent in each of the 500 postures. The overlaid lines are the full extent at half-maximum contours for the wild-isolate strains and for uncoordinated mutants. The distributions for the wild isolates and uncoordinated strains are plotted separately in figure 4*b*. Consistent with expectations from the N2 results, the wild-isolate strains, which are known to move more persistently on food than N2, are highly compressible, whereas worms that transition slowly between postures tend to be less compressible (there are few points in the upper right quadrant of the distribution). Nonetheless, differences in activity do not explain all of the variation in compressibility that is observed between strains.

This variation is clear from the strains highlighted in figure 4*a* (red bars, mean ± s.e.). The Hawaiian isolate CB4856 is known to be more active than N2 and it is also more compressible. However, two other hyperactive strains with loss-of-function mutations in *cat-2* and *npr-1* are significantly less compressible than CB4856 (figure 4*c*). This suggests that, even though they move more persistently than N2, their locomotion is less stereotyped—more random—than that of CB4856.

These differences could be due to the use of N2-derived postures for all of the strains. This is a particular concern for uncoordinated strains that will adopt postures not seen in N2. We therefore re-derived postures for each of the strains individually and re-calculated their compressibility/duration histograms (electronic supplementary material, figure S8). We also re-calculated the histograms using 250- and 1000-posture chunks (electronic supplementary material, figure S8). The conclusions about relative compressibility are not altered in either case.

## 5. Discussion

### 5.1. Hierarchical structure in behaviour

The task of finding relevant behavioural motifs from a long string of postures is analogous to the task of finding genes in unannotated genomic data. However, unlike the situation in genomics, we do not yet have a ‘behavioural code’ that could guide the search. Instead, we take a more general heuristic approach to finding meaningful sequences inspired by the minimum description length principle. When we compress sequences of worm postures, we generate a hierarchical structure, but one that does not show a very high degree of nesting. Instead, the repeat structure of worms' spontaneous locomotion is characterized by short motifs that are used repeatedly but not normally in the identical context. In this sense, worms' spontaneous locomotion on food is more like a novel than a poem or song with a chorus (electronic supplementary material, figure S3). This is consistent with previous results using *n*-gram frequencies in worm locomotion. There is a small number of frequently used *n*-grams and a much larger set of rare *n*-grams [18]. The structure we identify through compression suggests that the set of rare sequences is large enough to break up the repeated use of frequent patterns and to prevent the emergence of highly nested ‘patterns of patterns’.

A hierarchically organized action selection can lead to repetitive patterns in sequences [23], but the fact that a hierarchical representation can be constructed from a flat sequence does not necessarily imply that the underlying generating process is hierarchical. Instead, the nested structures we detect are best thought of as candidate behavioural units that may serve as hypothesized motifs for further study. Conversely, while there is more structure in worm behavioural sequences than in the corresponding shuffled data (figure 1*c*), we cannot rule out the presence of a deeper hierarchy in the underlying neural control. We would underestimate hierarchical structure if the output of a putative high-level command were implemented differently at the postural level because of environmental heterogeneity. That is, if different posture sequences were to be used because of different local conditions despite the same overarching command.

The organization of locomotion could be clarified by comparing patterns of behaviour with patterns of neural activity by imaging [24–28] and thermo- and optogenetic perturbation [15,24,29,30]. Experimental manipulation of modular behavioural units was recently used to uncover a hierarchy of actions in grooming flies [23]. A parallel model of action selection based on a suppression hierarchy was sufficient to reproduce the gross pattern of behaviours. In this case, the hierarchy of actions had a very simple structure in which activation of a higher behaviour suppressed the performance of the lower actions in the sequence. Dawkins referred to this kind of hierarchy as a ‘peck order’ [2] to distinguish it from more general control hierarchies that can have a complex branching structure. For complex hierarchies, even detecting the behavioural modules to probe may be more difficult. If modules can be identified and controlled, inferring the underlying control structure in the more complex case may be aided by using c-grams as candidate patterns to explore in more detail.

### 5.2. Rare but relevant motifs

Compared with the total set of unique *n*-grams, the c-grams that are identified by hierarchical compression are a much smaller subset. In the case of worms in different environmental conditions, from the total set of unique *n*-grams only 0.3% were identified as c-grams. These proved to be a diverse set of behavioural motifs that were informative for comparisons between worms in different environments even though they were discovered on a per worm basis without reference to the environments the worms were in. Hierarchical compression can thus serve as a pre-processing step in behavioural comparisons that will make it possible to apply behavioural motif analysis to the large behavioural databases that are increasingly being created through high-throughput phenotyping pipelines [3,4,8,31].

### 5.3. Compressibility as a quantitative phenotype

Compression provides a new measure for phenotyping that may give insights into mutant and wild-isolate differences. It was previously known that most wild isolates are faster on average than the laboratory strain N2, but we have found that this difference does not account for the differences we see in compressibility. For example, strains with a loss-of-function allele of the neuropeptide receptor gene *npr-1* show many of the phenotypes that are associated with wild isolates including increased speed, a shift in collective behaviour towards aggregation, as well as growth and pathogen avoidance [32,33]. However, we find that *npr-1* mutant behaviour is less compressible than the wild-isolate strains, including the well-studied Hawaiian strain CB4856. In other words, although they move persistently, their locomotion is more random than the wild isolates, as are the less persistent N2 worms.

The majority of mutant strains show patterns of locomotion that are less compressible (more random) than the wild-isolates. Ranked in terms of compressibility, the 17 wild isolate strains have a median rank of 301 out of a total of 337 strains that were analysed and a maximum rank of 250. Compressibility is related to predictability, and being too predictable, especially in response to sensory stimulation, can be deleterious in some circumstances; a fact that is strikingly demonstrated by tentacled snakes preying on fish [34]. Unpredictability is also likely to be important for worms as recent work has demonstrated that ongoing network activity increases behavioural variability above the level predicted by sensory noise [35]. Furthermore, a degree of randomness is an important element of *C. elegans* search strategies [36–38]. Nonetheless, during directed locomotion, the most efficient gait is likely to be repetitive, and so we speculate that the high compressibility of wild isolates reflects a selective pressure for efficient locomotion and that the more random locomotion observed in N2 is due to a relaxation of this pressure in a laboratory environment. Laboratory domestication is known to have occurred in N2 based on the analysis of other phenotypes [39,40]. Regardless of the ultimate cause, behavioural compressibility is a novel quantitative phenotype that is different between N2 and CB4856 and that is not explained by loss of *npr-1* function. It therefore presents an opportunity to explore the genetics of this behavioural difference using recombinant inbred lines derived from these strains [41–45].

### 5.4. Hierarchical compression beyond worms

Compressibility is a general measure that can be applied to the behaviour of any organism that can be tracked and discretized or converted to a series of labels by other means. The Nevill–Manning compressive heuristic has already been applied to human motion capture data [46–48] and our approach could be readily applied to an ethogram derived either manually or automatically for any organism, including humans. This last possibility is worth considering, because some human diseases affect locomotion (e.g. Parkinson's) and stereotypy (e.g. schizophrenia [49]) and compressibility might provide a simple scalar measure to quantify or even diagnose variation in a medically relevant phenotype.

## Supplementary Material

Supplementary figures

## Data accessibility

The latest version of the code used for compression and to generate the figures in the paper can be found at https://github.com/aexbrown/Behavioural_Syntax. Worm crawling data are available at http://wormbehavior.mrc-lmb.cam.ac.uk/.
