# Supplementary material for: Hierarchical compression of Caenorhabditis elegans locomotion reveals phenotypic differences in the organization of behaviour
Source: J R Soc Interface. 2016 Aug;13(121):20160466. doi: 10.1098/rsif.2016.0466 (PMC5014070; doi:10.1098/rsif.2016.0466)
Supplement: Supplementary figures [file rsif20160466supp1.pdf]

A

Sequence: 1211212212112122121

New rule: 3 → 121

Sequence: 332233223

New rule: 4 → 3223

Sequence: 344

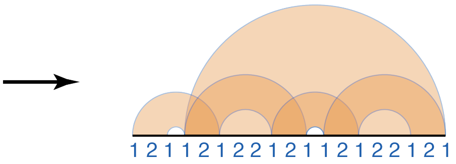

B

Iteration 1

Sequence: 1211212212112122121

unique *n*-grams with at least 2 occurances (no overlaps)	Counts	Savings *WN - (W + 1 + N)*
11	2	-1
12	7	4
21	7	4
22	2	-1
112	2	0
121	5	6
122	2	0
211	2	0
212	4	4
221	2	0
1121	2	1
1211	2	1
1212	2	1
1221	2	1
2112	2	1
2121	2	1
2122	2	1
2212	2	1
11212	2	2
12112	2	2
12122	2	2
12212	2	2
21121	2	2
21221	2	2
22121	2	2
112122	2	3
121121	2	3
121221	2	3
122121	2	3
211212	2	3
212212	2	3
1121221	2	4
1211212	2	4
1212212	2	4
2112122	2	4
2122121	2	4
11212212	2	5
12112122	2	5
12122121	2	5
21121221	2	5

Iteration 2

Sequence: 332233223

unique *n*-grams with at least 2 occurrences (no overlaps)	Counts	Savings *WN - (W + 1 + N)*
22	2	-1
23	2	-1
32	2	-1
33	2	-1
223	2	0
322	2	0
332	2	0
3223	2	1
3322	2	1

Iteration 3

Sequence: 344

unique *n*-grams with at least 2 occurances (no overlaps)	Counts	Savings *WN - (W + 1 + N)*
N/A	N/A	N/A

Most Frequent

Longest

Most Compressive

Fig. S1: Sequences considered by a brute force version of the compression algorithm. (A) Summary of the algorithm and graphical representation of compressive sequences. (B) All non-overlapping *n*-grams are counted. Only *n*-grams that occur at least twice in the sequence are included here (an *n*-gram that occurs only once can never compress the sequence because it takes

as many characters in the dictionary as in the original sequence and requires the creation of a new symbol). Potential candidate sequences that would be replaced by different heuristics are highlighted, including the most frequent, longest, and most compressive. The counts and savings (total reduction in the number of characters after all occurrences in the original sequence are replaced with the new symbol) for each candidate sequence on shown. The savings can be negative which means that the size of the combined dictionary and compressed sequence will be larger than the uncompressed sequence. In the first iteration, there is a single sequence {1, 2, 1} which gives the maximum compression when replaced. However, ties are possible. In the second iteration, both {3, 2, 2, 3} and {3, 3, 2, 2} are tied for the most compressive sequence (in this case they are also the longest repeated n-grams). In the case of ties, we take the sequence that occurs first in the sorted list of n-grams, not necessarily the first to appear in the sequence, which is why {3, 2, 2, 3} is added to the dictionary in this case. The algorithm terminates when no further savings can be achieved, in this case that is because there are no more repeated n-grams, but it would also occur if there were only 3-grams with counts of 2 remaining since these would lead to zero savings when replaced.

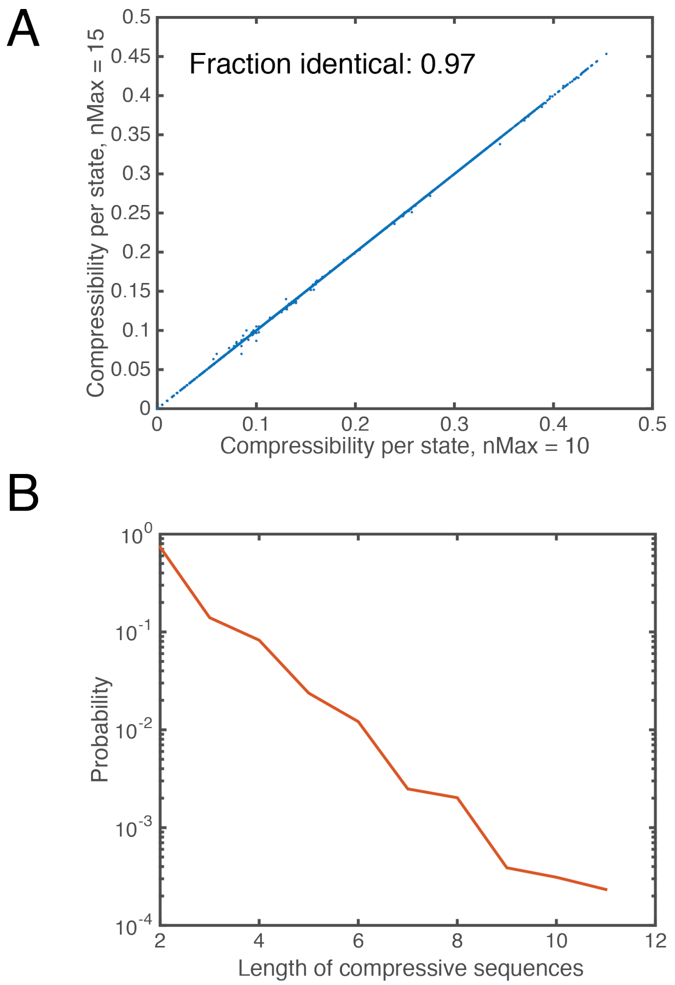

Fig. S2: Increasing the maximum length of *n*-gram considered during compression has little effect on the observed compressibility. (A) For 200 worms each recorded for 15 minutes, the algorithm proceeded identically in 97% of cases. For those cases where a difference was observed, the difference was small. (B) The reason is that the probability of a sequence being the most compressive in a given iteration decreases exponentially with length. In the 200 worms, 11-posture compressive sequences were rare and 12-posture compressive sequences were not observed.

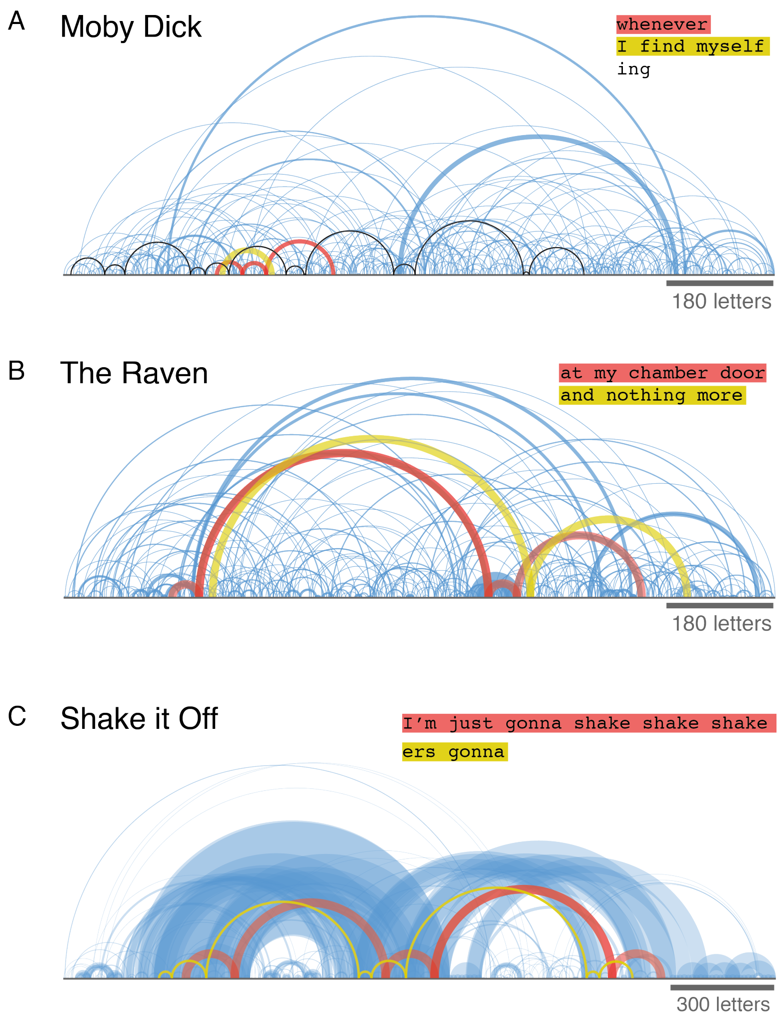

Fig. S3: Arc diagrams show repeats in texts with increasing structure. In all cases, punctuation and spaces were removed and case was ignored. (A) The first 1200 characters of Moby Dick by Herman Melville. Prose includes some repetition, but usually only at the level of words and short phrases. Three uses of 'whenever' and two of 'I find myself' are highlighted. To find a more frequent repeat that occurs throughout, it is necessary to go to shorter structures such as the part of a word 'ing' which is shown as the black arcs. (B) The first 1200 characters of The Raven by Edgar Allan Poe. Wider arcs corresponding to longer repeats are visible. The relationship between 'at my chamber door' and 'and nothing more' are clear in the highlighted arcs. (C) Shake it Off by Taylor Swift shows strong repeat structure. The third use of the chorus is slightly different from the previous two and so the repeat is not perfect. 'ers gonna' is an interesting c-gram because it is used in two phrases: 'Haters gonna hate' and 'Players gonna play'. The arc

diagram shows that both uses have an invariant structure with respect to ‘I’m just gonna shake shake shake.”

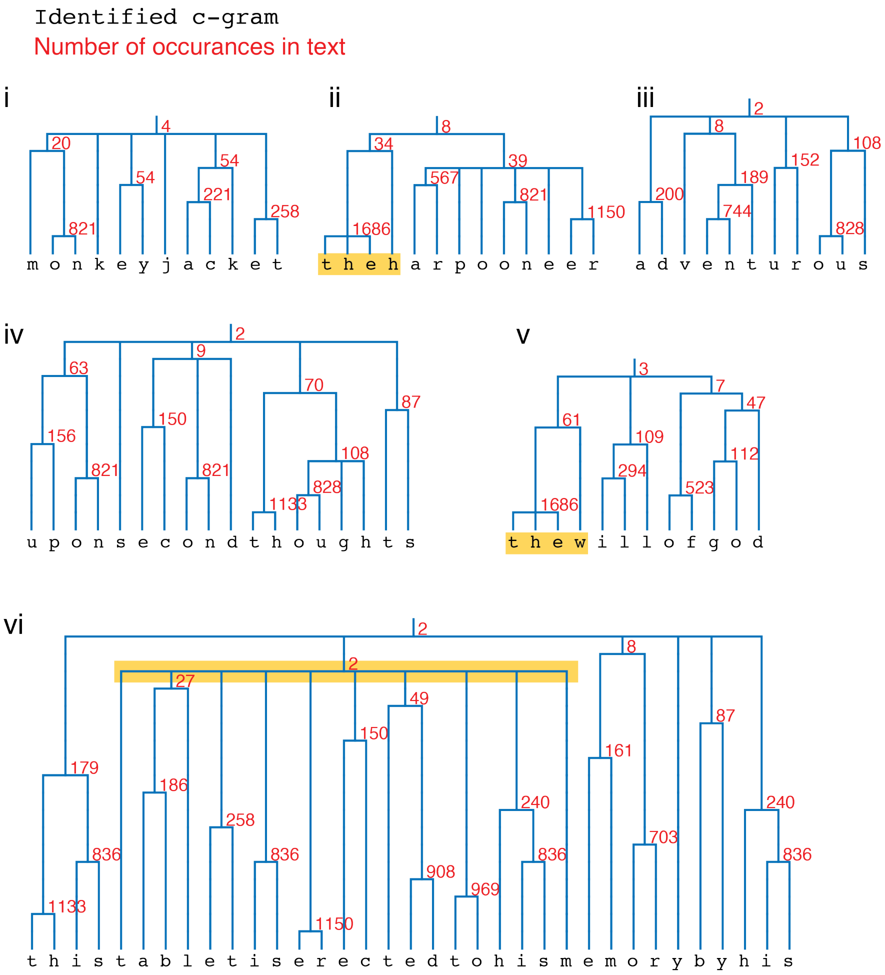

Fig. S4: Repeated phrases found as c-grams in Herman Melville's Moby Dick are related to themes of the novel. The repeated phrases are related to the themes of the novel. The inferred hierarchy gives insight into the Compressive heuristic. Not surprisingly, 'the' is found to be the most compressive sequence overall. However, because it is so frequent, it often gets associated with suffix letters and therefore breaks other words in the hierarchy. Two examples are highlighted in yellow in ii and iv. Because 'will' only occurs 34 times, 'thew' is more compressive and is included in an earlier round of compression, effectively blocking the introduction of 'will' because of the greedy nature of the algorithm. iv is the longest repeat in the novel and illustrates a feature of language that is not commonly observed in worm locomotion. The highlighted branch of the dendrogram shows how 10 small branches merge in one step to form a phrase. While similar one-step merging of many short c-grams is observed in worm sequences (e.g. the pause in Fig. 1D), it is rare.

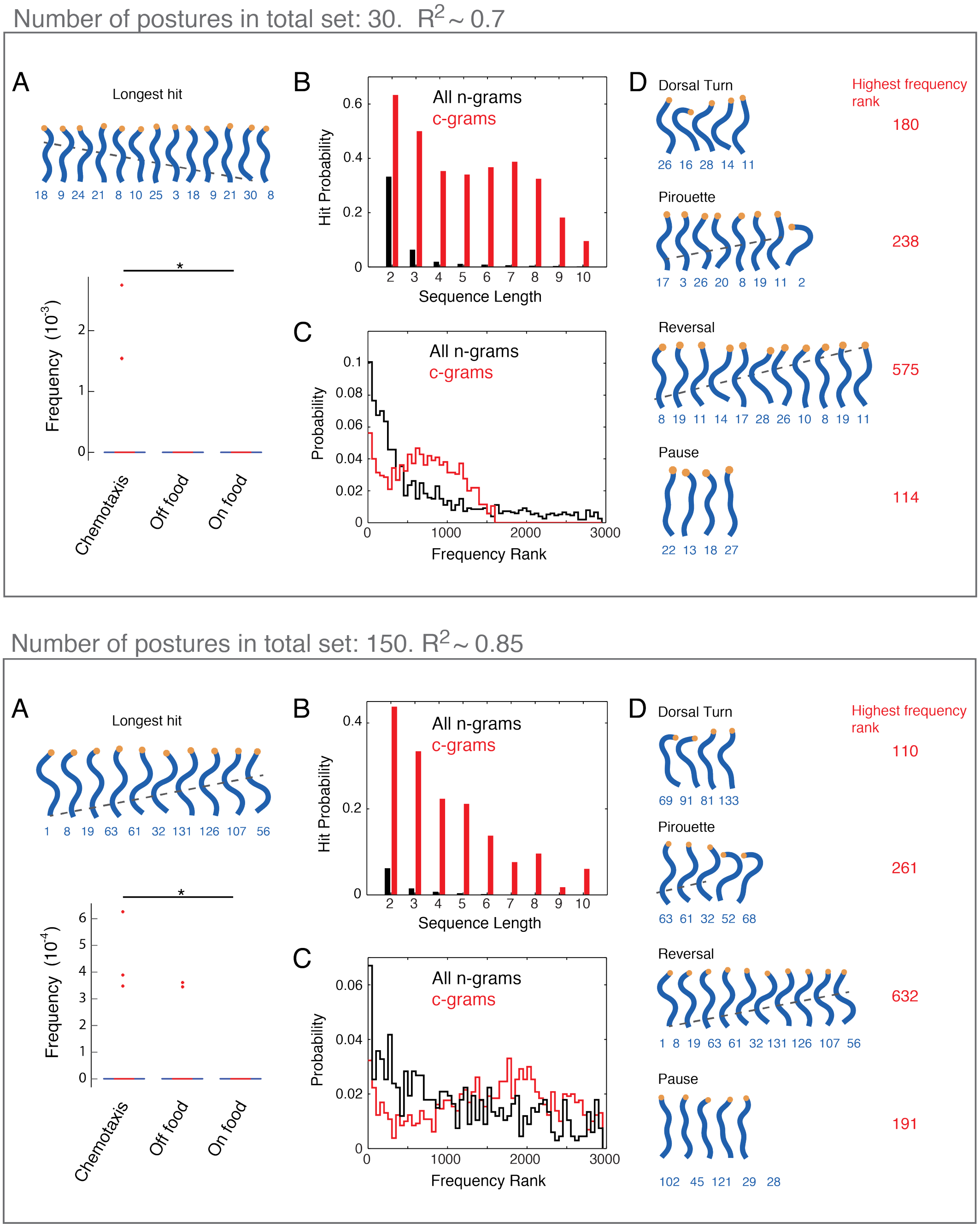

Fig. S5: Changing the number of postures used to represent the worm behaviour changes the results quantitatively, but not qualitatively. The R^2 value in gray for each posture number set indicates the average quality of the fit between the set of template postures and the original worm posture data from tracking. Reducing the number of postures leads to a less accurate fit but more compact representation and vice versa.

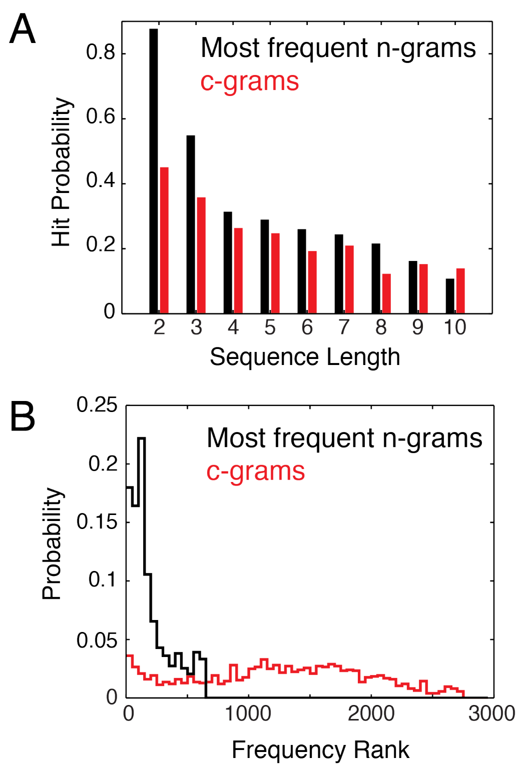

Fig. S6: (A) The hit rate of *n*-grams is increased if only the 5 most frequent *n*-grams of each length from each worm are considered. However, this comes at the cost of ignoring rare sequences (B).

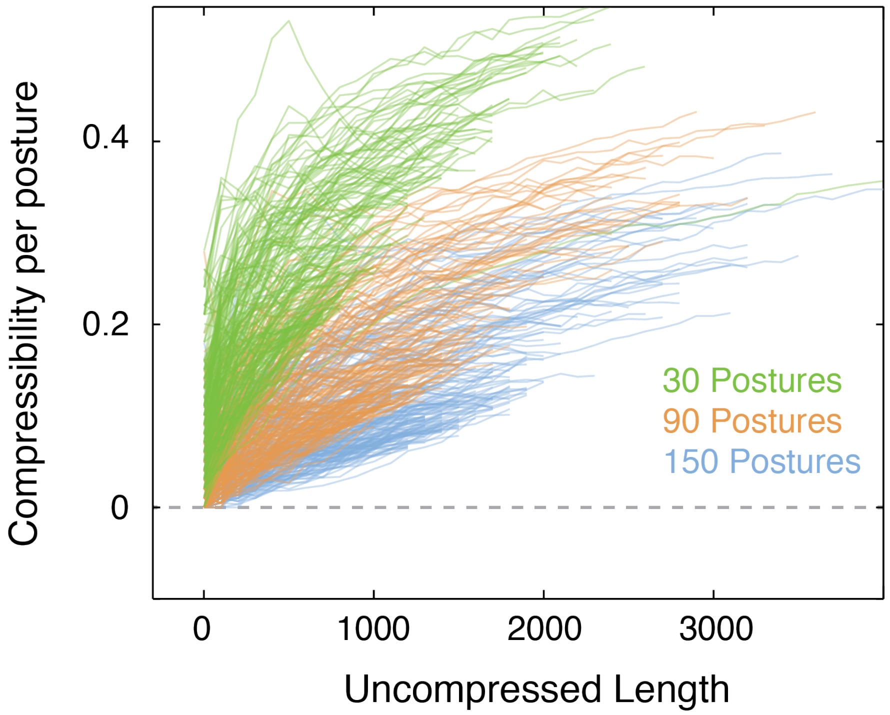

Fig. S7: Increasing the total number of template postures used to represent behaviour decreases compressibility, in line with expectations.

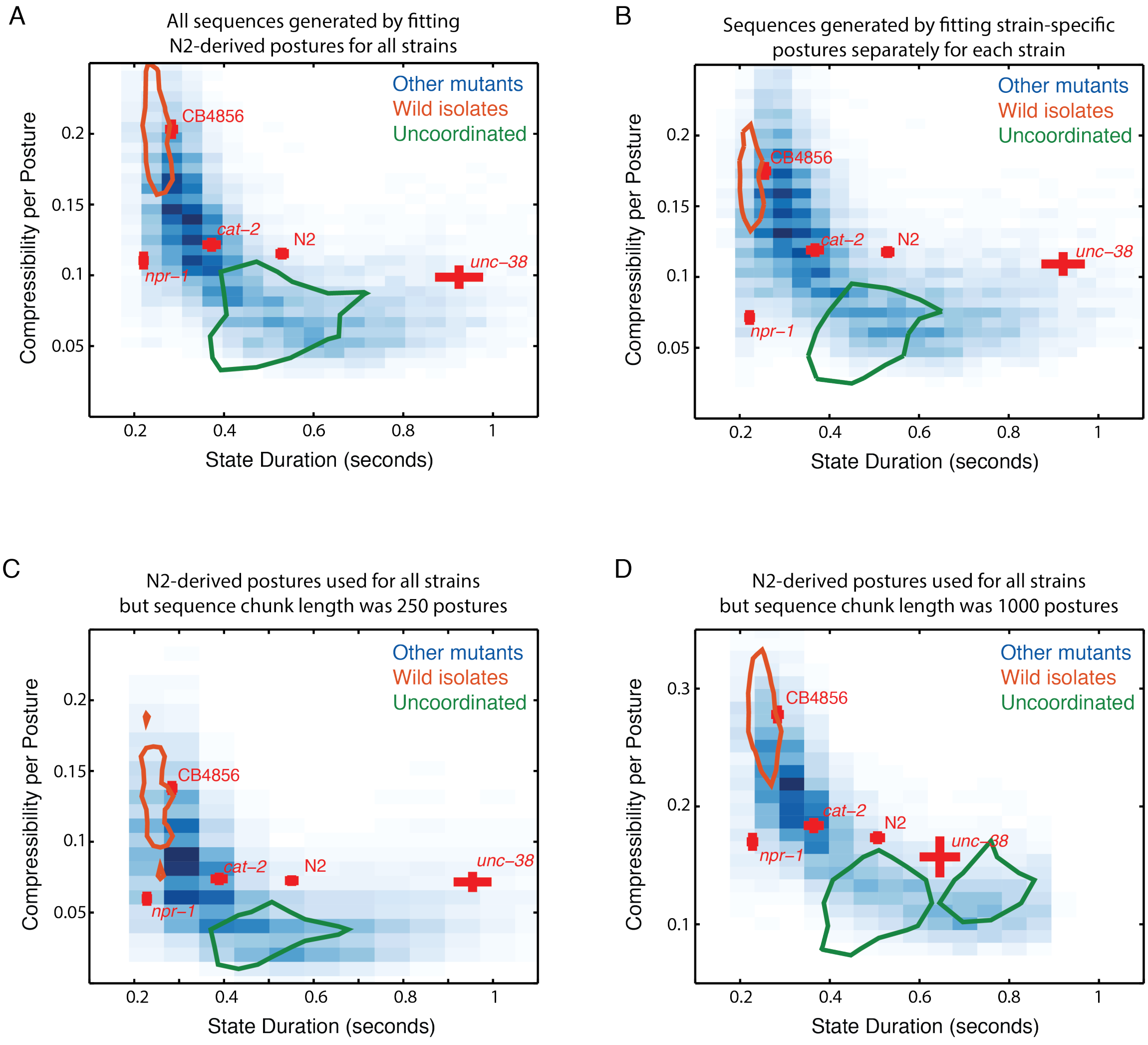

Fig. S8: 2-dimensional compressibility/state duration histograms. (A) The histogram using N2-derived postures and a chunk size of 500 postures, this is a reproduction of Fig. 4A in the main text. (B) The same histogram but using compressibilities calculated using template postures re-derived for each strain. *npr-1* becomes less compressible in this case, but this only enhances the difference between it and the wild isolates. (C) This histogram was generated using N2-derived postures but shorter 250-posture chunks to calculate the compressibility. Compressibility is always lower, but the relationships between strains are similar. (D) Same as A and C, but using 1000-posture chunks for the calculation. Compressibility increases, but again the relative differences are similar. There is a truncation of the duration axis and a resulting shift in the *unc-38* duration because for slow-moving strains, some 15 minute videos include fewer than 1000 postures and were not included.
